# Study protocol of a phase II clinical trial of oral metformin for the intravesical treatment of non-muscle invasive bladder cancer

**DOI:** 10.1186/s12885-019-6346-1

**Published:** 2019-11-21

**Authors:** Remco J. Molenaar, Jons W. van Hattum, Iris S. Brummelhuis, Jorg R. Oddens, C. Dilara Savci-Heijink, Egbert R. Boevé, Saskia A. van der Meer, J. Fred Witjes, Michael N. Pollak, Theo M. de Reijke, Johanna W. Wilmink

**Affiliations:** 1 Department of Medical Oncology, Cancer Center Amsterdam, Amsterdam University Medical Centers, University of Amsterdam, Amsterdam, the Netherlands; 2 Department of Urology, Cancer Center Amsterdam, Amsterdam University Medical Centers, University of Amsterdam, 1105 AZ Amsterdam, The Netherlands; 3 Department of Urology, Jeroen Bosch Ziekenhuis, Henri Dunantstraat 1, 5223 GZ ‘s-Hertogenbosch, The Netherlands; 4 Department of Medical Pathology, Cancer Center Amsterdam, Amsterdam University Medical Centers, University of Amsterdam, Amsterdam, the Netherlands; 5 Department of Urology, Sint Franciscus Hospital, Kleiweg 500, 3045 PM Rotterdam, The Netherlands; 6 Department of Urology, Radboud University Medical Center, Geert Grooteplein Zuid 10, 6525 GA Nijmegen, The Netherlands; 7 Departments of Oncology and Medicine, McGill University, McIntyre Medical Building, 3655 Sir William Osler, Montreal, Quebec, H3G 1Y6 Canada; 8 Segal Cancer Centre, Jewish General Hospital, 3755 Chemin de la Côte-Sainte-Catherine, Montreal, Quebec, H3T 1E2 Canada

## Abstract

**Background:**

Non-muscle-invasive bladder cancer (NMIBC) is the most common neoplasm of the urinary tract and requires life-long invasive surveillance to detect disease recurrence. Currently, there are no effective oral therapies that delay disease recurrence or progression. We recently demonstrated that in mice, metformin accumulates unchanged in the urine. Urothelial cells are exposed to metformin concentrations ~ 240-fold higher than in serum. This was effective in the treatment of mouse bladder cancer models.

**Methods:**

We describe the protocol of a multi-centre, open-label, phase II clinical trial of metformin in up to 49 evaluable patients with intermediate-risk NMIBC with the aim to determine the overall response to administration of oral metformin for 3 months on a marker tumour deliberately left following transurethral resection of multiple, papillary NMIBC tumours. All patients will receive metformin orally at doses up to 3000 mg per day. Metformin treatment will start within 2 weeks following transurethral resection of all tumours except one marker lesion. After 3 months of metformin treatment, the effect of metformin on the marker lesion is evaluated by cystoscopy and biopsy under anaesthesia. Residual tumour, if present at this evaluation, will be resected. In case of complete disappearance of the marker lesion, the former tumour area will be biopsied. The primary outcome is the complete response rate of the marker lesion, as determined by decentralised scoring of pre- and post-treatment cystoscopy images by expert independent urologists. Secondary outcomes are the partial response rate, overall safety of metformin and the duration of the time to recurrence.

**Discussion:**

Preclinical studies show the potential role of oral metformin treatment in the management of NMIBC. It could offer an alternative to current adjuvant intravesical treatment. If positive, the reported results of this study could warrant further phase III trials to compare the efficacy of metformin against current treatments of intravesical installations with chemotherapy or Bacillus Calmette-Guérin (BCG).

**Trial registration:**

This trial is registered in ClinicalTrials.gov under NCT03379909.

## Background

Non-muscle-invasive bladder cancer (NMIBC) is the most common neoplasm of the urinary tract [1]. Despite current treatments, i.e. transurethral resection followed by adjuvant intravesical treatment with Bacillus Calmette-Guérin (BCG) immunotherapy or mitomycin C chemotherapy, it is still associated with a high risk of recurrence and/or progression [2]. The need for life-long invasive surveillance and treatment results in a high patient burden and puts NMIBC among the most expensive cancers to treat on a per-patient basis, which represents a major public health challenge. Therefore, the development of a safe, low-cost, and orally active drug for preventing recurrence and progression of NMIBC is a priority in urologic oncology.

There were ~ 3750 new cases of NMIBC in The Netherlands in 2018. The incidence is rising, from 10 new cases per 100,000 person-years in 1990 to 24 new cases per 100,000 person-years in 2018 (www.cijfersoverkanker.nl). Bladder cancer is the fifth most prevalent malignant disease for men and the tenth among women, and occurs mainly in the middle-aged and elderly [1, 3, 4]. NMIBC accounts for 70–80% of bladder cancers at first presentation. Stages Ta-T1 make up the majority of cases, with 5–10% diagnosed as primary carcinoma-in-situ (CIS), while a small percentage of patients presents with both. Recurrence rates following transurethral resection of the bladder tumour (TURB) vary from 30% for a single papillary tumour to over 90% in case of multiple tumours [4]. CIS has been viewed as both an aggressive neoplasm with high probability of invasion and alternatively as having limited biologic capacity to invade. The unpredictability of both papillary and CIS bladder tumour progression has led to the widespread use of adjuvant intravesical therapy, both in controlling existing tumours (therapy) and in preventing tumour recurrence (prophylaxis) [5].

### Metformin as an anti-cancer drug

Metformin is a widely used, inexpensive and safe drug that is first-in-line for the treatment of diabetes mellitus type 2 (T2DM). Substantial laboratory evidence supports the hypothesis that metformin has antineoplastic activity [6]. Moreover, epidemiologic studies have suggested that metformin reduces the occurrence, recurrence and progression of NMIBC in patients with T2DM, as compared with T2DM patients not treated with metformin [7–10]. While there are many ongoing trials of metformin for various indications in oncology, metformin has not been able to improve patient survival so far in any type of cancer [11–13]. One explanation could be that metformin fails to accumulate to a sufficient concentration in tissues to exert sufficient antineoplastic effects. In support of this, many in vitro studies show direct antiproliferative actions of metformin at millimolar concentrations [14–20], but after oral administration of metformin the highest attainable serum concentrations in vivo are in the micromolar range [11, 21].

Metformin is excreted unchanged in the urine and mice studies have shown that following oral dosing, the urothelium is exposed to urine metformin concentrations that are ~ 240-fold higher than in the circulation [18]. This resulted in improved survival, reduced urinary tract obstruction and reduced bladder weight of NMIBC-bearing mice [18]. In humans, metformin is also excreted unchanged in the urine, where concentrations are > 100-fold higher compared to the serum [22]. Thus, from a pharmacokinetic perspective, NMIBC is particularly interesting for clinical trials to evaluate metformin as an antineoplastic agent. Molecularly, metformin inhibits bladder cancer cell proliferation by various mechanisms, such as activation of AMPK and inhibition of mTOR [14–18] and inhibition of STAT3-mediated pathways [19, 20]. These anti-cancer properties of metformin were observed both *in vitro* [14] and intravesically in vivo [16–20] and both as metformin monotherapy [18] as well as in combination with other anti-cancer therapeutics, such as cisplatin [16] and gefitinib [17]. Of note, metformin markedly prolonged the survival of mice that were genetically engineered to develop bladder cancer [18].

### Leaving a marker lesion to study NMIBC response

A problem in the management of NMIBC is the frequency and multiplicity of tumour recurrence and the primary concern is, therefore, the efficacy of adjuvant therapy in delaying or preventing this recurrence. Previous studies have demonstrated that the ablative activity of intravesical therapy relies upon the response of Ta/T1 tumours remaining in the bladder, but these are confounded by the presence of multiple tumours of indeterminate number, size and characteristics [23]. The response rate observed for any drug can, therefore, vary because of this heterogeneity. Thus, it is recognized, though not universally accepted, that a marker lesion procedure is the most objective way of conducting phase II studies using novel agents for the treatment of Ta/T1 NMIBC lesions, before initiating large-scale registration trials [23]. All agents that have proven to be beneficial in prophylactic studies also have an ablative effect leading to a complete response of the marker lesion in up to 50–60% [23]. Several points concerning the safety of a marker lesion study can be made. First, prognostic factor analyses performed by the European Organisation for Research and Treatment of Cancer (EORTC) and the Medical Research Council (MRC) have shown that the likelihood of progression to muscle invasive bladder cancer (MIBC) in patients with multiple low-grade, intermediate risk NMIBCs is 0–2% and treatment with unproven agents is, therefore, acceptable [24]. Second, in a systematic review including 23 studies leaving a marker lesion in more than 1200 patients, 7 patients (0.6%) showed progression to muscle invasive bladder cancer (MIBC) [23]. However, progression was only seen in studies that included patients with a high-risk prediction score for NMIBC progression. Although it is unknown whether the progressed patients indeed had high-risk NMIBC, it is very likely that marker lesion experiments are safe in patients with low-, and intermediate-risk NMIBC. Third, if NMIBC is considered a pan-urothelial disease then clearly tumour cells or their precursors remain in the bladder after a visibly complete resection. In clinical studies in patients with a solitary Ta/T1 tumour, 5–20% of patients had a tumour at 4 weeks following a supposedly complete resection [25, 26]. A marker lesion procedure requires 2 cystoscopy procedures within a relatively short timeframe (one before [which is also performed in the standard of care] and one at the end of study treatment) and the extra cystoscopy can thus aid in the resection of any additional or persisting tumours that were not recognized at the first cystoscopy procedure. Fourth, before embarking on a larger-scale phase III trial, this approach prevents that many patients are exposed to a possible inferior treatment.

### Hypothesis and outlook

To summarise, the investigation of metformin in patients with NMIBC is warranted by several *in vivo* studies [16–20] and epidemiological evidence [7–10] demonstrating that metformin reduces bladder cancer risk, recurrence and progression in diabetic patients. Critically, it is likely that NMIBC cells are exposed to efficacious metformin concentrations because metformin accumulates unchanged in the urine and this exposes urothelial cells to metformin concentrations that are ~ 240-fold higher than in the serum [18]. Combined, these data provide a strong rationale for a phase II clinical trial of high-dose oral metformin in the treatment of NMIBC. We hypothesise that oral metformin administration results in therapeutic metformin concentrations in the urine and that this leads to tumour response of a NMIBC marker lesion that was deliberately left in situ*.*

## Methods/design

### Overall study design

This ‘*Phase II study of oral metformin for intravesical treatment of non-muscle-invasive bladder cancer*’ (TROJAN) study is a nonrandomized, open-label, multi-centre phase II clinical trial with oral metformin treatment in patients with intermediate-risk NMIBC. Patients will be enrolled at four hospitals in The Netherlands (the Amsterdam UMC in Amsterdam, the Jeroen Bosch Ziekenhuis in ‘s-Hertogenbosch, the RadboudUMC in Nijmegen and the Sint Franciscus Hospital in Rotterdam). This is a marker lesion study, meaning that in NMIBC patients undergoing TURB, a marker lesion will be deliberately left in the bladder. Patients will be treated with metformin for 3 months. Subsequently, the response on the marker lesion will be determined in a second TURB and the marker lesion is resected or (in case of a complete response) the area of the former marker lesion will be biopsied. After the second TURB, patients may choose to continue with metformin treatment.

### Objectives

#### Primary objective

The primary objective is to determine the complete response rate after administration of oral metformin for 3 months. This is assessed using a marker tumour deliberately left following transurethral resection of NMIBC lesion(s).

#### Secondary objectives

- To determine the partial response rate following administration of oral metformin for 3 months in a marker tumour deliberately left following transurethral resection of NMIBC lesion(s);
- To determine the time to recurrence;
- The safety of metformin in this population;
- Quality of life during metformin treatment using two questionnaires (SF-36 and EORTC NMIBC-24).

#### Exploratory objectives

- The effect of metformin on tumour biology markers in biopsies or tumour resection tissue taken before and after study treatment;
- The pharmacokinetic analysis of metformin levels in urine and serum;
- In case of promising results: a preliminary economic evaluation to estimate potential cost savings that could be achieved using metformin in NMIBC patients.

### Trial end points

#### Primary end points (outcomes)

The primary outcome is the complete response rate after 3 months of treatment with metformin. Evaluable patients are those who have received at least 500 mg metformin twice daily for one week and who undergo a cystoscopy for marker lesion removal. Primary study parameters are:

- Complete response: complete disappearance of the marker lesion, as confirmed by negative biopsy at the marker lesion site, the absence of new tumours at other sites and a negative bladder washout cytology;
- No response: marker lesion persistence or the appearance of new lesions with stage T1 or less.

#### Secondary end points (outcomes)

- The partial response rate is determined by the proportion of patients that have a ≥ 30% decrease of the sum of the longest perpendicular marker lesion diameters;
- The duration of the time to recurrence of NMIBC, in which patients who have continued metformin treatment after the study will be compared to those who did not. Patients will be followed for a maximum duration of 5 years. In case of a complete response; the time to recurrence is the duration of the complete response. In case of a partial response, no response or progressive disease, this is the time to recurrence after resection of the marker lesion;
- The number of grade 1–4 and grade 5 (fatal) NCI Common Terminology Criteria for Adverse Events Version 4.03 (CTCAE) events during treatment with metformin. All patients will be evaluable for toxicity from the time of their first treatment with metformin.

#### Exploratory end points (outcomes)

- The immunohistochemical expression of tumour biology markers such as AMPK and TSC2 before and after metformin treatment.
- Trough metformin levels in serum and metformin levels in a sample of urine collected over 12 h in samples taken after 6 weeks and after 3 months of metformin treatment. These levels will be pharmacokinetically analysed and correlated to the marker lesion response data and the patient’s toxicity data.

### Participants

In brief, this trial will enrol 49 eligible patients with NMIBC. All inclusion and exclusion criteria are listed in Table 1. The sample size is based on a power analysis described under Statistical methods.

**Table 1:** Inclusion and exclusion criteria

Key inclusion criteria	
• Age ⩾18 years. • Patients with primary or recurrent multiple histologically confirmed Ta or T1 (non-muscle invasive), G1 or G2 (low-grade) urothelial carcinoma of the bladder with no evidence of carcinoma in situ. • Patients must have at least 2 lesions, but no more than 10. • The resected lesions must contain detrusor muscle to confirm a Ta/T1 disease. • All visible lesions must be completely removed by transurethral resection at entry to the study, except for an untouched marker lesion measuring 0.5–1.0 cm in its greatest dimension. • Bimanual examination immediately following transurethral resection under anaesthesia should be carried out and no mass should be felt. • Adequate renal function (creatinine < 150 μmol/L and/or an eGFR > 60 ml/L). • Adequate liver function (bilirubin < 1.5 times upper limit of normal, ALAT or ASAT < 2.5 the upper limit of normal). • Eligible patients must be fully informed of the investigational nature of the study and written signed informed consent must be obtained prior to any study specific investigations. • Mentally, physically, and geographically able to undergo treatment and follow up.	
Key exclusion criteria	
• Patients having muscle-invasive disease (stage T2 or greater) or CIS. • Patients with grade 3 (high-grade) tumours. • Patients with diabetes mellitus receiving metformin or having received metformin in the past 6 months. • Patients who have received intravesical treatment (chemotherapy or immunotherapy) within the last 3 months. • Patients that are currently receiving other anti-cancer therapy. • Patients with existing urinary tract infection or recurrent severe bacterial cystitis. • Patients that need to be treated with a transurethral catheter. • Patients with urogenital tumours with histology other than urothelial carcinoma (i.e., squamous cell or adenocarcinoma) or with urothelial carcinoma involving the upper tract or the prostatic urethra. • Patients with a history of other primary malignancy (other than squamous or basal cell skin cancers or cone biopsied CIS of the uterine cervix or prostate carcinoma treated curatively with normal PSA values at inclusion) in the last five years. • Patients with active, uncontrolled impairment of the renal, hepatobiliary, cardiovascular, gastrointestinal, urogenital, neurologic or hematopoietic systems that, in the opinion of the investigator, would predispose to the development of complications from the administration of metformin. • Patients who are using loop diuretics, cimetidine, ranitidine, cetirizine, trimethoprim, vandetanib, kinidine and/or anti-HIV medication (due to drug interactions), for which no reasonable alternative is available. • Women who are pregnant or lactating. Individuals of reproductive potential may not participate unless agreeing to use an effective contraceptive method for themselves and/or their sexual partner. • Patients with ECOG-WHO performance status of 3 or 4. • Patients with a known history of alcohol abuse. • Patients with a known hypersensitivity to metformin. • Patients who in the investigator’s opinion, cannot comply with provisions of the protocol or do not understand the nature of the study.	

Abbreviations: *CIS* carcinoma in situ, *ECOG* Eastern Cooperative Oncology Group, *eGFR* estimated glomerular filtration rate, *HIV* human immunodeficiency virus, *PSA* prostate-specific antigen, *WHO* World Health Organisation

### Dose of study drugs and dose adjustments

All patients will receive metformin orally in a starting dose of 500 mg once daily and, if well tolerated, this will be increased to 500 mg twice daily in the second week, 1000 mg twice daily in the third week and, with a good renal function, eventually to a maximum of 3000 mg per day. This highest metformin dose is reserved to those with an estimated glomerular filtration rate (eGFR) of ≥60 mL/min to reduce the risk of lactic acidosis. Based on toxicity data from patients with T2DM, we expect that 2550 mg of metformin (850 mg three times daily) is a realistic maximum tolerable dose in most patients with an eGFR of ≥60 mL/min [27]. This dosing schedule conforms with the metformin label from the US Food and Drug Administration in the treatment of T2DM and the purpose of the stepwise dose increase is to reduce gastro-intestinal side effects [28]. Doses will be reduced for haematological and other adverse events. Dose adjustments are to be made according to the system showing the greatest degree of toxicity. Examples of metformin dose adjustments due to frequently occurring adverse events are shown in the Additional file 1**.**

### Toxicity monitoring and adverse events

Adverse events will be graded using the CTCAE version 4.03. The major adverse effects of metformin, which may limit the maximum tolerable dose, are of gastro-intestinal nature, such as nausea, vomiting, diarrhoea, abdominal discomfort and loss of appetite. A rare but serious adverse event that can occur during metformin treatment is lactic acidosis [29]. This life-threatening condition is caused by accumulation of metformin. Risk factors include a combination of old age, doses over 2000 mg per day and renal impairment, either due to chronic renal impairment or due to acute renal failure as the result of dehydration, shock or the intravenous administration of iodinated contrast agents. The estimated prevalence of lactic acidosis is < 5 cases per 100,000 patient years [30].

### Study visits

Patients with NMIBC will visit their hospital of inclusion once for TURB and additional eligibility screening (see in- and exclusion criteria). Once enrolled in the study, patients will undergo a study visit after 6 weeks, when blood will be drawn and urine will be collected for analysis of hematologic, hepatic, renal, and chemistry parameters and for pharmacokinetic analyses. After 3 months, these procedures will be repeated and patients will undergo a cystoscopy and resection of their marker lesion if still present or a biopsy of the marker lesion site. Specifics for each study visit are provided in Table 2. Patients will have a telephone appointment with their study physician before every metformin dose increase. For most patients, these phone calls will thus be scheduled after weeks 1, 2, and 3.

**Table 2:** Timeline, study treatment, study visits and medical procedures

Required Investigations	Prestudy	Telephone calls	6 weeks after start treatment	3 months after start treatment (end of treatment)
Written informed consent	X			
Demographics	X			
Overall medical history	X			
ECOG/WHO performance status	X	X	X	X
Adverse events		X	X	X
EORTC QLQ-NMIBC24	X		X	X
SF-36 quality of life	X		X	X
Medication diary		X	X	X
Physical:				
Physical examination	X		X	X
TURB	X			X
Haematology:				
Complete blood counts	X		X	X
PT, APTT	X			
Clinical chemistry:				
Renal function	X		X	X
Liver function	X		X	X
Serum glucose	X		X	X
HbA1c	X			X
Insulin	X			X
IGF-1	X			X
IGF binding protein-3	X			X
Other Investigations:				
Metformin concentration in serum			X	X
Metformin concentration in urine			X	X
Urinalysis	X		X	X
Pathological review of biopsies	X			X

Abbreviations: *ECOG* Eastern Cooperative Oncology Group, *IGF* insulin growth factor, *WHO* World Health Organisation

### Therapy response assessment

The cystoscopic assessment of marker lesions is generally considered to be relatively insensitive to inter- and intra-observer heterogeneity. However, in order to pursue the most unbiased and reproducible analysis possible, cystoscopic video images of the marker lesion before and after metformin treatment will be stored and independently scored by three independent urologists that are blinded to the timing of the image acquisition (pre- or post-treatment). Differences in opinion will be discussed and, if necessary, solved by a fourth independent urologist. The urologist will judge whether there is a complete response (disappearance of the marker lesion), partial response (at least a 30% decrease of the sum of the longest perpendicular marker lesion diameters) or no response. Partial responses will be determined using the 8-mm span of the cutting loop for reference. We will perform immunohistochemical staining of tumour biology markers such as AMPK and TSC2 to investigate the molecular response of the marker lesion to metformin.

### Pharmacokinetics

Pharmacokinetics of metformin are monitored to evaluate a relationship between drug exposure, toxicity, and/or efficacy. Blood samples will be taken at several time points during the study for the determination of the respective plasma levels. The half-life of metformin is ±6 h [31], which means that with daily dosing the plasma level of metformin reaches a steady-state concentration within two days. Pre-dose plasma samples (i.e. prior to study medication ingestion) will be taken on day 43 (after 6 weeks) and day 92 (after 3 months). Patients will collect their urine during 12 h on day 42 and day 91, of which a sample will be taken in which the metformin level will be determined.

### Statistical methods

The patient sample size in the clinical trial (*n* = 49) is based on a two-part trial design:

- Part 1: Initially, 13 patients will be treated. If no responses are seen in the first 13 consecutive, evaluable patients, the probability of a response rate of ≥30% in the overall NMIBC patient population is < 1% (type II error) and the trial shall be terminated.
- Part 2: If one or more responses are seen in the first part, the study will continue until the enrolment of 49 evaluable patients is met. This allows to establish a response rate of 50% with a power of 80%, an α error of 5%, and to reject with the same power a response rate of < 30%.

According to statistical review, the analysis after part 1 is not an interim analysis and additional correction is not necessary. For the secondary endpoint concerning partial responses, the tumour dimensions captured by decentralised measurements from cystoscopy images before and after the metformin treatment will be compared using the paired samples *t*-test.

### Data management, auditing and access

Source data from the trial will be locally stored and entered in electronic case report forms. Based on the guidelines by the NFU (Dutch Federation of University Medical Centres) the risk of this study was qualified as ‘moderate’. According to this, a ‘minimal intensive auditing’ is advised, which will be performed by an independent clinical research associate. Besides this clinical research associate, only the investigators are allowed access to the source data. As part of informed consent, patients will be informed as to the strict confidentiality of their patient data, but their medical records may be reviewed for trial purposes by clinical research associates. This clinical trial does not have a data and safety monitoring board (DSMB).

### Informed consent

All patients will be informed by the investigator(s) concerning the aims of the study, the possible adverse events, the procedures, the possible hazards to which he/she will be exposed, who has access to their patient data and what provisions were made for compensating those who suffer harm from trial participation. It will be emphasised that the participation is voluntary and that the patient is allowed to refuse further participation in the protocol whenever he/she wants. Refusing study participation will not influence the patient’s subsequent regular medical care. Documented informed consent must be obtained for all patients included in the study before they are enrolled in the study.

### Harms

(Serious) adverse events and (serious) adverse drug reactions will be collected and recorded throughout the study period, starting at the first day of treatment through 1 month after the last dose of investigational product in accordance with Good Clinical Practice guidelines as described by the International Conference on Harmonisation (ICH-GCP). It will be left to the investigator’s clinical judgment to determine whether an adverse event is related to study treatment and of sufficient severity to require the subject’s removal from treatment or from the study. A subject may also voluntarily withdraw from treatment.

### Economic evaluation

The need for invasive treatment (with BCG or mitomycin C) and life-long surveillance (with a cystoscopy every 3 to 12 months) puts NMIBC among the most expensive cancers to treat on a per-patient basis and it represents a major public health challenge. This is augmented because NMIBC is a common cancer. Of note, metformin is off patent and, therefore, inexpensive: one year treatment of metformin 3000 mg per day costs €20.25 in the Netherlands. In this preliminary economic evaluation, we discriminate between two scenarios that do not need to be mutually exclusive:

1. *Metformin may decrease the need for intravesical adjuvant instillations with BCG or mitomycin C.* The costs of a single dose of 40 mg mitomycin C (€164.54) and BCG (€161.21) are similar. These costs are added to the costs for the instillation procedure itself (~€1.300, source: passantentarieven Amsterdam UMC, AMC 2019). In the first year after TURB, patients with intermediate-risk NMIBC need a total of 15 intravesical instillations with BCG or mitomycin C, with a preference for BCG [4]. Thus, the total costs per patient for adjuvant treatment with BCG in the first year after TURB are ~€22,000 (15 * (€1300 + €161.21)). In the case that oral metformin maintenance therapy renders intravesical instillations with BCG obsolete in 50% of NMIBC patients (corresponding with an expected 50% response rate in this clinical trial), this will reduce the annual medical costs of adjuvant NMIBC treatment with ~€44 million (50% * 4000 * (€22,000 – €20.25) - 50% * 4000 * €20.25).
2. *Metformin may be effective in improving local control rates and prolonging response durations of NMIBC so that extended intervals between cystoscopic surveillance become safe.* A cystoscopy costs ~€775 and if a biopsy on the outpatient clinic needs to be performed and pathologically analyzed, this costs ~€420 extra. These costs increase dramatically if a TURB needs to be performed: €5.063,20. The average interval between follow-up with cystoscopies is, approximately, six months [4]. Therefore, the total costs per patient are ~€1550 per year (2 * €775). In the case that metformin treatment can safely double the interval between cystoscopies and biopsies from every six months to every year in 50% of NMIBC patients (corresponding with an expected 50% response rate in this clinical trial), this will reduce the annual medical costs of NMIBC surveillance in the Netherlands by ~€11.6 million (50% * 32000 * (€775 – €20.25) - 50% * 32,000 * €20.25).

In view of the early stage of evidence generated in our proposed study (phase II), an economic evaluation seems rather premature. However, in case the results suggest that metformin is effective in improving local control rates and prolonging response duration of NMIBC, we will develop a preliminary economic model to estimate long-term benefits and costs associated with treatment of NMIBC.

## Discussion

The design of the study protocol we present here comes with several strengths; To the best of our knowledge, this is the first clinical trial that investigates the treatment of non-muscle invasive bladder cancer with oral metformin. The marker lesion procedure allows intra-patient assessment of tumour response to metformin before exposing patients to this new treatment modality in a large phase 3 study. Some limitations of the study design are; the fact that the marker lesion procedure may be a hurdle for rapid patient inclusion. The ideal application of metformin in non-muscle invasive bladder cancer may not be in ablating lesions but in chemoprevention of recurrences, a treatment indication for which a marker lesion study is not the appropriate design. In non-muscle invasive bladder cancer, chemoprevention rather than chemoresection is sought for as replacement of the current standard of bladder installations with chemotherapeutic drugs of BCG.

Overall there is a solid foundation of preclinical studies that warrants a clinical trial to explore the potential role of metformin in the management of NMIBC.

If positive, this phase II trial could legitimate phase III trials in intermediate- and possibly even high-risk NMIBC patients. In the best-case scenario, these trials will show that adjuvant metformin is more effective than adjuvant BCG/mitomycin and will, therefore, render intravesical BCG/mitomycin treatment obsolete (economic evaluation, scenario 1) and also extend the safe interval between cystoscopic surveillance (economic evaluation, scenario 2), in which case the potential savings can be added. In other scenarios, metformin may be non-inferior to BCG/mitomycin, but extending the interval between cystoscopic surveillance may not be safe. In that case, only the savings from scenario 1 apply. In brief, the nature of the proposed oral metformin treatment offers various cost-saving opportunities compared with the current standard of care for NMIBC.

## Supplementary information

**Additional file 1.** Dose reduction in case of side effects. Dose reduction in case of diarrhoea.

## Data Availability

Data sharing is not applicable to this article as no datasets were generated or analysed during the current study.

## Abbreviations

AMPK: Adenosine monophosphate kinase
b.i.d.: Bis in die, two times a day
BCG: Bacillus Calmette-Guérin
CCMO: Dutch national competent authority
CTCAE: Common Terminology Criteria for Adverse Events
ETC: Electron transport chain
ICH-GCP: International Conference on Harmonisation – Good Clinical Practica
NMIBC: Non-muscle-invasive bladder cancer
q.d.: Quaque die, one a day
T2DM: Diabetes mellitus type II
TCA cycle: Tricarboxylic acid cycle
TSC2: Tuberous sclerosis complex 2
TURB: Transurethral resection of the bladder
