# Supplementary material for: Study protocol of a phase II clinical trial of oral metformin for the intravesical treatment of non-muscle invasive bladder cancer
Source: BMC Cancer. 2019 Nov 21;19:1133. doi: 10.1186/s12885-019-6346-1 (PMC6873510; doi:10.1186/s12885-019-6346-1)
Supplement: Supplementary file 1 — Additional file 1. Dose reduction in case of side effects. Dose reduction in case of diarrhoea. [file 12885_2019_6346_MOESM1_ESM.docx]

SUPPLEMENTARY FILE 1.

Dose reduction in case of side effects

| NCI-CTCAE grade (CTC 4.0) | Percent of full dose |
| --- | --- |
| 0-2 | 100 |
| 3 | 50 or hold |
| 4 | Hold |

Dose reduction in case of diarrhoea

| NCI- CTCAE  grade  (v4.0) | Study Drug Modification | Management |
| --- | --- | --- |
| Grade 1 | None | Consider loperamide (4 mg at first onset), followed by 2 mg every 2-4 hours until the patient is diarrhoea free for 12 hours. |
| Grade 2 | Dose reduction of metformin is necessary if diarrhoea persists over 48-72 hours despite optimal medical management. | Loperamide (4 mg at first onset), followed by 2 mg every 2-4 hours until the patient is diarrhoea free for 12 hours. |
| Grade 3 | Interrupt metformin until resolution to grade < 2, and restart at next reduced dose | Rehydration if necessary. Loperamide (4 mg at first onset), followed by 2 mg every 2-4 hours until the patient is diarrhoea free for 12 hours. |
| Grade 4 | Discontinue metformin. | Rehydration if necessary. Loperamide (4 mg at first onset), followed by 2 mg every 2-4 hours until the patient is diarrhoea free for 12 hours. |
